# Intelligent Detection and Analysis of Polycyclic Aromatic Hydrocarbons Based on Surface-Enhanced Raman Scattering Spectroscopy

**DOI:** 10.1155/2022/8330702

**Published:** 2022-02-02

**Authors:** Qian Zhang, Bowen Chen, Fazli Wahid, Wanyun Feng, Xuerou Chen

**Affiliations:** ^1^College of Chemistry and Chemical Engineerging, China West Normal University, Nanchong, Sichuan 637002, China; ^2^Yangtze Delta Region Institute, University of Electronic Science and Technology of China, Huzhou, Zhejiang 313099, China

## Abstract

Cycloaromatic hydrocarbons are a type of potentially hazardous chemicals that are widely present in the environment and pose a serious threat to human health. However, the traditional research methods for their detection process are cumbersome, the detection cycle is long, and the sensitivity is low. In response to the above problems, this article combines the molecular fingerprint information characteristics of surface-enhanced Raman scattering technology to simulate the four polycyclic aromatic hydrocarbons of pyrene, anthracene, phenanthrene, and trichenium and quantitative detection of cyclic aromatic hydrocarbons and four kinds of polycyclic aromatic hydrocarbon mixtures. The experimental results show that the PAHs based on SERS have the advantages of higher sensitivity and high selectivity, which verifies the accuracy and feasibility of the method in this article.

## 1. Introduction

In recent years, more and more people have begun to pay attention to the importance of supramolecular interactions in nature. This type of role is mainly based on the use of artificially designed selective recognition substrates and target substances for molecular recognition [1]. The supramolecular system itself can be used in many fields such as drug sustained release and molecular sensing, and it can be used in combination with other technologies to take into account the applications of the two systems. Some researchers combine surface-enhanced Raman technology with some supramolecular bodies, such as calixarene [2] and cyclodextrin [3] for qualitative and quantitative detection and analysis. SERS technology has a more attractive aspect than Raman technology in terms of enhancement factor exceeding 1010. It is widely used in chemistry with its own high sensitivity [4], high selectivity [5], and no fluorescence [6] and the fields of biosensing and detection. Combining with supramolecular systems can make up for the inability of SERS technology to be applied to molecules that have no physical or chemical interaction with the SERS substrate, inducing these molecules to combine with supramolecular systems to approach the area where the electromagnetic field is enhanced to determine the SERS signal of the molecule [2, 3].

Polycyclic aromatic hydrocarbons are organic compounds containing two or more benzene rings or heterocyclic rings and their substitutions. These compounds are hardly soluble in water and easily soluble in organic solvents. They are produced by incomplete combustion of organic polymer compounds such as coal, petroleum, wood, and tobacco. They are the main environmental and food pollutants, and a considerable part of them are carcinogenic [7]. Therefore, the detection of polycyclic aromatic hydrocarbons has always been a hot direction of scientific research. The existing PAHs detection technologies include gas chromatography [8, 9], high-performance liquid chromatography [10], chromatography spectrometry [11, 12], thin-layer chromatography, and capillary electrophoresis [13], The advantages and disadvantages of immunoassays are shown in Table 1. Among them, the current mainstream detection methods for PAHs in the world are gas chromatography-mass spectrometry (GC-MS) and high-performance liquid chromatography (HPLC).

The characteristics and applicability of these detection methods are different, and there are many problems in the use process, such as long detection cycle, low sensitivity, and high cost. Therefore, in this paper, the SERS model combining cyclodextrin and gold nanoparticles is used for qualitative I dentification and trace detection of polycyclic aromatic hydrocarbons. The complex system of four polycyclic aromatic hydrocarbons (pyrene, anthracene, phenanthrene, and naphthalene) mixture is selected, and the design is used. The SERS-enhanced model is used for qualitative identification and quantitative detection. Experiments show that this method can be applied to the detection of PAHs mixture systems and has a certain application potential.

## 2. Related Work

Polycyclic aromatic hydrocarbons are a type of serious chemical pollutants. How to detect them quickly and accurately is an important scientific problem. In this paper, cyclodextrin is selected as the host molecule and modified on the surface of the SERS substrate. With the high sensitivity of SERS technology and the high selectivity of cyclodextrin, a quick and easy detection method for organic pollutants such as polycyclic aromatic hydrocarbons is developed, to achieve the purpose of qualitative identification and quantitative detection.

### 2.1. Surface-Enhanced Raman Spectroscopy

In the mid-1970s, the VanDuyne research group and the Creighton research group summarized experimentally and theoretically and found that this is a regular phenomenon based on rough surfaces, which is called surface-enhanced Raman scattering (surface-enhanced Raman scattering) (SERS) effect, that is, when the sample molecules are adsorbed (or approached) on the rough surface of metal materials such as gold, silver, and copper, the Raman signal can be greatly enhanced, and the method is established based on this enhanced effect with surface selectivity, it is called surface-enhanced Raman spectroscopy [14, 15]. With the continuous development of nanomaterial technology, transition metal has also achieved SERS response. After continuous development, surface-enhanced Raman spectroscopy can realize single-molecule detection due to its high sensitivity; simple sample pretreatment, which can realize rapid on-site detection; good reproducibility, strong specificity, and advantages in environmental monitoring, food drug testing, biomedical analysis, etc. have been widely used [16, 17]. In surface-enhanced Raman spectroscopy, the Raman signal of the molecule to be measured is enhanced by exciting the local surface plasmon resonance (LSPR) of the substrate, so the magnitude of SERS enhancement mainly depends on the SERS substrate. However, the roughening of the substrate makes the preparation of the substrate a certain challenge. In the past few decades, in order to continuously improve the sensitivity, stability, and reproducibility of SERS detection, researchers have conducted a lot of research on SERS substrate materials [18, 19]. SERS technology requires the adsorption of analyte molecules onto the SERS substrate. After being adsorbed on the SERS substrate, the Raman signal of the analyte is enhanced. Unlike fluorescence, the spectral peaks obtained in SERS are narrower [20, 21].

The Raman shift characterizes the vibration characteristics of different groups in the molecule. Therefore, the Raman shift can be measured for qualitative and structural analysis of the molecule. Raman spectroscopy can be used for structural analysis of organic compounds. Due to different chemical environments, the Raman shifts of the same functional groups of different molecules have certain differences and will vary within a certain range, so it is convenient to distinguish various isomers, such as positional isomerism, geometric isomerism, cis-trans isomerism. For some groups, the Raman scattering signal is strong and the characteristics are obvious, which is also suitable for Raman determination. Raman spectroscopy can also be used for the study of polymer compounds, suitable for the determination of its geometric configuration, carbon chain skeleton or ring structure, and crystallinity. The polymer compound of the inorganic compound filler can be directly measured without separation. Raman spectroscopy is also an effective method for studying biological macromolecules, and it has been used to determine the structure of biochemical substances such as proteins, amino acids, carbohydrates, biological enzymes, and hormones. At the same time, Raman spectroscopy can study the composition, conformation, and interaction of biomolecules at extremely low concentrations close to the natural state. In addition, biological tissue sections such as eye lens, skin, and cancer tissue can be directly measured without complicated processing. Therefore, Raman spectroscopy has been widely used in biology and medical research.

### 2.2. Detection Methods and Progress of Polycyclic Aromatic Hydrocarbons

At present, for the detection of PAHs in the environment and food, high performance liquid chromatography (HPLC) and gas chromatography-mass spectrometry (GC-MS) are mostly used, which are two more popular methods. High-performance liquid chromatography is a common separation technology. The method is used to determine polycyclic aromatic hydrocarbons. Generally, dichloromethane is used as the solvent, methanol water or acetonitrile-water is used as the mobile phase, and single or mixed solutions buffers, etc. The mobile phase is input into a chromatographic column equipped with a stationary phase. After the components in the column are successfully separated, it enters the fluorescence and ultraviolet detectors for detection to realize the analysis of the sample. This method has high separation efficiency, good selectivity, and high detection sensitivity. But the shortcomings are also particularly obvious. The solvent consumption is large, the types of detectors are few, the price is expensive, and the cost is high, and it can only detect organics with high boiling points and poor thermal stability. Zhang Qian et al. [22] constructed a method for detecting the residues of polycyclic aromatic hydrocarbons in the soil, using rapid solvent extraction, solid phase extraction, and gel permeation chromatography to purify the method, using high-performance liquid chromatography ultraviolet fluorescence. The detector is connected in series, and the method conforms to the national standard of measurement. He Yan [23] and others used high school liquid chromatography with ultraviolet and fluorescence detectors to determine 16 kinds of polycyclic aromatic hydrocarbons in the air, which is simple and quick to operate.

The advantage of gas chromatography-mass spectrometry (GC-MS) method is that the method can present good specificity and discrete monitoring ability in ion mode. GC-MS is different from high-performance liquid chromatography and is only suitable for analytes with poor volatility and good thermal stability. Gas chromatography has the advantages of high sensitivity and strong qualitative analysis reliability. Using gas as the mobile phase can achieve rapid equilibrium with the stationary phase, thereby achieving high precision and rapid analysis in a short time. Mass spectrometry, GC-MS has high resolution and lower detection limit for PAHs, which is recommended in many PAHs analysis methods. Liquid chromatography can realize the separation of nonvolatile substances and trace polar compounds. Although liquid chromatography cannot provide complete information of individual PAHs and alkyl-substituted PAHs like gas chromatography, it is coupled with fluorescence or mass spectrometry detectors. When used, the detection of a single PAHs in a complex mixture can be achieved. HPLC is a high-pressure, high-efficiency, high-speed, high sensitivity, and wide-ranging analysis technology. HPLC can significantly improve the sensitivity of analysis by combining it with a fluorescence detector (FLD) or mass spectrometry detector. Due to the fluorescent properties of PAHs, HPLC is usually used in combination with FLD. ERS amplifies the Raman scattering signal of the target compound through the surface plasmon resonance phenomenon of metal nanoparticles and nanopatterned structures under the synergistic effect of electromagnetic and chemical effects. Due to the high hydrophobicity of PAHs, the affinity to the metal SERS active surface is low, resulting in low detection sensitivity of SERS. Functionalizing the surface of the substrate to increase the affinity for PAHs molecules is an important way to solve this problem. Capillary electrophoresis (capillary electrophoresis, CE) is an electrophoretic separation and analysis method that uses a capillary as a separation channel and a high electrostatic voltage field as the driving force. Capillary zone electrophoresis (CZE) is one of the most conventional CE methods. A CZE method based on an optimized cyclodextrin (CD) modification has been developed. This method is easier, faster, and more efficient. Be selective.

### 2.3. Application and Progress of Detection of Polycyclic Aromatic Hydrocarbons Based on Surface-Enhanced Raman Technology

At present, the detection methods for polycyclic aromatic hydrocarbons mainly include gas chromatography, liquid chromatography, high performance liquid chromatography and capillary electrophoresis. However, these methods require complicated pre-processing and time-consuming and labor-intensive, which brings difficulties to the general application of the method. Therefore, it is urgent to develop a fast, sensitive and simple detection method for qualitative identification and quantitative detection of PAHs. With the development of nano-preparation and characterization technology, SERS spectroscopy has made significant developments in both the basic principle research and the field of substrate preparation. Today, the sensitivity and reproducibility of many new SERS substrates have reached practical standards. However, for those molecules that do not have substituent groups that can interact with precious metal substrates, it is often necessary to use other modification methods to modify the SERS substrate to perform SERS detection on these molecules. For the SERS detection of PAHs, the methods of modifying the SERS substrate can be roughly divided into five categories:

Long-chain alkanes modified SERS substrates SERS humic acid modified SERS substrates; calixarene modified SERS substrates; amethyst dication-modified SERS substrates and mercapto-substituted cyclodextrin-modified SERS substrates. Regardless of the modification method of the SERS substrate, the general idea is to use the interaction between the PAHs molecules and the surface modification molecules of the SERS substrate to bring them close to the surface of the modified substrate, and then reach the enhanced area of the enhanced substrate surface, so as to complete the modification. SERS detection. Several SERS substrate modification methods will be introduced and compared below. The specific PAH molecules detected by several modified substrates and the corresponding detection limits are listed in Table 2.

Surface-enhanced Raman spectroscopy still has some problems in the detection of PAHs based on SERS substrates modified by supramolecular chemistry. The long-chain alkane-modified SERS substrates use alkyl substituents to modify the noble metal SERS substrates, mainly using the hydrophobic interaction between PAHs and long-chain alkanes. This method can obtain high detection sensitivity, but the detection selectivity is poor. Humic acid is a macromolecular organic acid composed of aromatics and various functional groups, which has good physiological activity and functions such as absorption, complexation, and exchange. The detection of PAHs on the humic acid-modified SERS substrate is also based on the hydrophobic interaction between the two, so it also encounters the problem of poor selectivity. The interaction between the viologen dication-modified SERS substrate and PAHs, in addition to the hydrophobic effect, also includes the *π*-*π* interaction between the aromatic rings. This method helps to improve the adsorption selectivity of the SERS substrate to the measured substance. The use of supramolecular chemistry to modify the surface of SERS substrates is an important development in the detection of PAHs. Future research is expected to focus on two aspects. On the one hand, further improve the reproducibility of the SERS substrate, combined with stoichiometric methods. On the other hand, it can be combined with other analytical techniques, especially separation techniques, to make the qualitative and quantitative analysis of SERS spectroscopy more perfect.

## 3. Experiment and Analysis

The 60 nm gold sol was purchased from BB International with a concentration of 3.4 × 10^−11^M. Chemical reagents such as anthracene, pyrene, phenanthrene, and pyrene were purchased from Wako, Japan. The experimental water is tertiary deionized water. First, the gold sol was mixed with sulfhydryl-substituted cyclodextrins of different concentrations overnight, and then the ethanol stock solution of polycyclic aromatic hydrocarbons was diluted with water to the desired concentration and mixed overnight. Finally, the polycyclic aromatic hydrocarbons entering the cyclodextrin cavity are centrifuged with different centrifugal speeds.

### 3.1. Experimental Instrument

The Raman spectrometers used in the experiment were all from Photon Design, and the 488 nm argon ion laser was used as the excitation light source. The power of the laser finally reaching the sample is about 1 mW. All Raman and spectra adopt Rubberband method for baseline correction.

Pyrene is a light yellow (pure product is colorless) solid aromatic compound. Its molecular formula is C_16_H_10_. It can also be oxidized to obtain 1,4,5,8-naphthalene tetracarboxylic acid is used to make fuels, synthetic resins, and engineering plastics; in addition, it can be acylated with> 1 winter pyrene to obtain vat dyes.

Anthracene is a light yellow needle-like crystal with light blue fluorescence (pure band purple fluorescence), so it has another name “flashing crystal”, and its molecular formula is C_14_H_10_.

Phenanthrene is a white flaky crystal whose molecular formula is C_14_H_10_. Phenanthrene and anthracene are isomers, so they have many similarities with anthracene.

Naphthalene is a colorless flake crystal with a special smell, and its molecular formula is C_10_H_8_. Naphthalene has the characteristics of being volatile and easy to sublime, so it must be kept in a sealed container.

### 3.2. Experimental Results and Analysis

**Figure 1:**
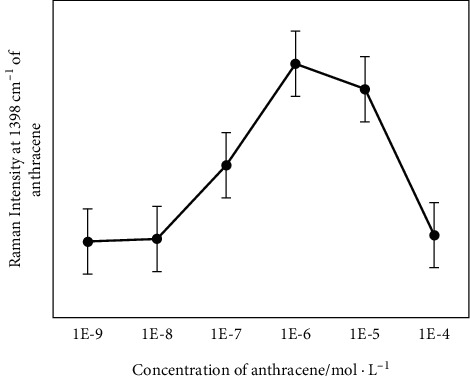
SERS intensity of an anthrancene marker band at 1398 cm^−1^ versus the concentration of CD-SH.

In this experiment, gold nanoparticles modified with sulfhydryl-substituted cyclodextrin were used to detect polycyclic aromatic hydrocarbons. In order to judge the influence of the coverage of the sulfhydryl-substituted cyclodextrin on the surface of the gold nanoparticles on the detection of polycyclic aromatic hydrocarbons, we selected different concentrations of supramolecular bodies from 10^−4^ to 10^−9^M and mixed with the same amount of gold sol. Then 10-5M anthracene molecule is used as the detection molecule, and SERS detection is performed on it. The peak intensity at 1398 cm^−1^ is selected as the research object, and the curve of the peak intensity at 1398 cm^−1^ is drawn with the change of the concentration of sulfhydryl-substituted cyclodextrin. The result is shown in Figure 1. As the concentration of supramolecular host increases, the intensity of the SERS peak of anthracene first increases and then decreases. This phenomenon is related to the optimal coverage of sulfhydryl-substituted cyclodextrin on the surface of gold nanoparticles. The experimental result is that the best SERS enhancement effect is achieved when the concentration of cyclodextrin is 10^−6^M.

In order to theoretically verify the above experimental results, we have carried out theoretical calculations on the surface coverage of the system. First, we assume that the best SERS enhancement effect is achieved when the gold nanoparticle surface is completely covered. From formulas (1)–(3) and the size of the gold nanoparticles and the size of the cyclodextrin, we can roughly calculate that the concentration when the thiol-substituted cyclodextrin completely covers the gold nanoparticles is 12 × 10^−6^M. The theoretical calculation results are in good agreement with the experiment.

(1) $$\begin{array}{c} S_{\text{gold}}=S_{S-\beta CD}=4\pi r^{2}, \end{array}$$

(2) $$\begin{array}{c} \frac{S_{\text{gold}}}{S_{S-\beta CD}}=\frac{C_{S-\beta CD}}{C_{\text{gold}}}, \end{array}$$

(3) $$\begin{array}{c} C_{S-\beta CD}=\left(\frac{S_{\text{gold}}}{S_{S-\beta CD}}\right)*C_{\text{gold}}. \end{array}$$

Among them, S_gold_ and S_S-*β*CD_ are the surface areas of gold nanoparticles and sulfhydryl-substituted cyclodextrin, respectively, and C_gold_ and C_S-*β*CD_ are the molar concentrations of gold nanoparticles and sulfhydryl-substituted cyclodextrin, respectively. When the concentration of sulfhydryl-substituted cyclodextrin was lower than 1 × 10^−6^M, the SERS signal of PAHs increased as the amount of cyclodextrin adsorbed on the surface of gold nanoparticles increased. This is because more mercapto-substituted cyclodextrin means that more cyclodextrin PAHs host-guests are adsorbed on gold nanoparticles, and more PAHs enter the enhanced area of gold nanoparticles. In turn, a stronger SERS signal can be obtained. The best effect is when the concentration of sulfhydryl-substituted cyclodextrin is equal to 1 × 10^−6^M. When the concentration of sulfhydryl-substituted cyclodextrin is greater than 1 × 10^−6^M, these molecules are combined with PAHs, so that excess cyclodextrin molecules and PAH molecules will be removed during centrifugation. As a result, the strength is reduced.

## 4. SERS Qualitative Identification and Quantitative Detection of PAHs

In order to achieve the best results in the detection of PAHs, we used unequal centrifugal speeds to enrich PAHs, and then select optimum centrifugal speed. The 10^−5^M anthracene molecule was used as the detection molecule, and other experimental conditions were unchanged. The centrifugal speed was from 1000 to 2500 rpm/s. After centrifugation, the sediment was retained to remove the supernatant, and the operation was repeated twice after washing with water.

### 4.1. Quantitative Analysis Components of PAHs by SERS Spectroscopy

**Figure 2:**
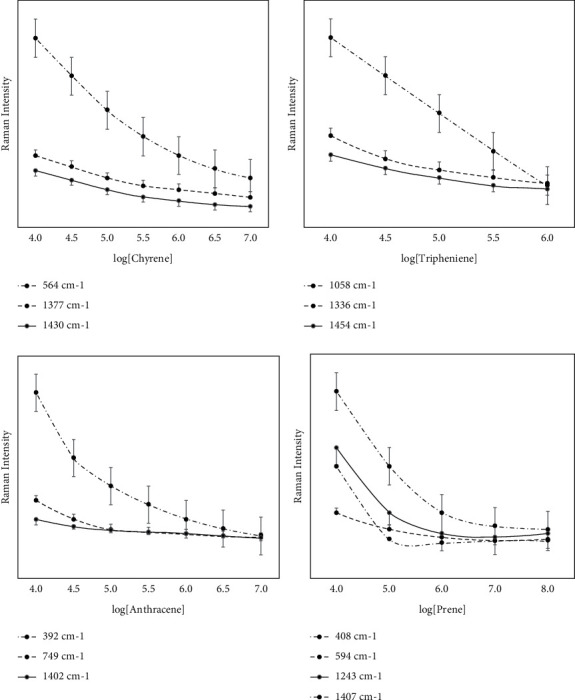
SERS intensity at discriminant peaks versus concentrations for anthracene, pyrene, chrysene, and triphenylene with CD-SH functionalized AuNP.

We have done comparative experiments in the previous section to prove that this SERS signal is indeed derived from the supramolecular modified SERS substrate we designed instead of the precious metal itself. For the four types of PAHs we studied, the measured SERS signals are in good agreement with each solid.  Figure 2, respectively, represents the dependence of the peak intensity of several characteristic peaks selected from each SERS spectrum of the four PAHs, anthracene, pyrene, phenanthrene, and phenanthrene, as a function of concentration. It can be seen from the figure that the SERS intensity of the four polycyclic aromatic hydrocarbons shows almost the same trend as the concentration decreases. In addition, the detection limits for these four polycyclic aromatics are 100, 10, 100, and 1000 nM. Using this modification method, the detection limits for anthracene and pyrene molecules are increased by 100 times.

From Figure 2, we can intuitively compare the detection capabilities of the four polycyclic aromatic hydrocarbons. The relationship between the detection limits is pyrene > anthracene > triphthone > phenanthrene. This can be explained by the size matching effect between polycyclic aromatic hydrocarbons and the inner cavity of cyclodextrin. The inner cavity of cyclodextrin has the largest molecular size of benzene. It is difficult to enter the inner cavity of cyclodextrin. In addition, after centrifugation, there is almost no signal from the benzene molecule. For the pyrene molecule, it is likely to form a composite structure of subject-object-subject. This composite structure uses the pyrene molecule as a “molecular bridge” to connect two gold particles, which forms the “hot spot effect” of SERS to enhance the signal of the pyrene molecule. It can be inferred from this that the SERS effect obtained by using the SERS model is closely related to the size matching effect of the polycyclic aromatic hydrocarbon molecules and the cyclodextrin cavity.

### 4.2. SERS Qualitative Identification and Quantitative Detection of PAHs Mixture

In this paper, sulfhydryl-substituted cyclodextrin-modified gold substrates have been used to obtain SERS spectra with better signal to noise ratios for SERS detection of single polycyclic aromatic hydrocarbons. Next, continue to study the qualitative identification of the mixture of four PAHs. We can clearly distinguish the existence of each PAHs, and there are some new SERS peaks in the figure. On the one hand, we infer that these are due to the four PAHs, each of which competes to enter the cyclodextrin cavity, which makes the spectrum more complicated. On the other hand, when the concentration of PAHs decreases, the noise is relatively large, and some impurity peaks are more obvious. We fixed the concentration of the three polycyclic aromatic hydrocarbons to remain unchanged, changed the content of one of the polycyclic aromatic hydrocarbons, and used the cyclodextrin-modified SERS substrate designed in this experiment to perform SERS quantitative detection of the mixture system. We changed the concentrations of anthracene and pyrene molecules from 500 to 50 *μ*M to maintain the concentration of the other three polycyclic aromatic hydrocarbons at 10 *μ*M and extracted the peak intensity at 1539 cm^−1^, 1259cm^−1^, and 390 cm^−1^ as a function of concentration. The intensity of each peak of anthracene and pyrene in the mixture exhibits roughly the same law as the concentration changes, which proves that this method can be applied to the detection of polycyclic aromatic hydrocarbon mixture systems and has a certain application potential.

## 5. Conclusions

In this article, we designed an SERS substrate that combines supramolecular action, that is, a gold substrate modified by sulfhydryl-substituted cyclodextrin. This substrate has the characteristics of stability and long storage time, making it similar to polycyclic aromatic hydrocarbons that have no effect on gold. Of molecules are detected using SERS technology. The effect of the surface coverage of sulfhydryl-substituted cyclodextrin on the effect of gold nanoparticle surface on SERS and the effect of centrifugal speed on the results of SERS are discussed in detail. It has realized not only the quantitative detection of single components of pyrene, anthracene, phenanthrene, and benzophenone but also the quantitative detection of a variety of complex systems of polycyclic aromatic hydrocarbons. The experimental results show that the detection of PAHs based on SERS has the advantages of higher sensitivity and high selectivity, which verifies the accuracy and feasibility of this method. SERS-based PAH detection methods have promising applications in analytical chemistry and environmental science research, but these are mostly limited to basic and laboratory research. The real application of SERS in practical environmental science awaits further development of large-area-ordered SERS active substrates and further communication and cooperation between chemists and environmental scientists in this field.

## Figures and Tables

**Table 1:** Existing detection methods of PAHs and their advantages and disadvantages.

Detection method	Advantages	Disadvantages
Gas chromatography	High selectivity, high separation efficiency, high sensitivity, fast speed	Small application range, weak pertinence
High-performance liquid chromatography	Can analyze multiple compounds at the same time, good selectivity, high detection sensitivity	Solvent consumption is large, detectors have high separation efficiency, price is expensive
Chromatography and mass spectrometry	High separation efficiency, can determine the structural characteristics of the compound instrument	Expensive equipment, immature interface technology
Thin-layer chromatography	Simple, fast speed, intuitive	Low sensitivity, low separation efficiency
Capillary electrophoresis	High separation efficiency, simple operation	Sensitivity of existing detectors is not high
Immunoassay method	Strong pertinence, high sensitivity, low cost	Only one substance can be analyzed at a time, little information

**Table 2:** Detection limit corresponding to polycyclic aromatic hydrocarbon molecules.

Modification materials	Detection molecule	Detection limit/M
	Pyrene	10^–8^
Long chain alkanes	Naphthalene	10^–7^
	Phenanthrene	10^–7^
Viologendication	Pyrene	10^–9^
	Pyrene	10^–8^
Calixarene	Triphenylene	10^–9^
	Coronene	10^–10^
	Anthracene	10^–7^
Cyclodextrin	Pyrene	10^–8^
	Triphenylene	10^–6^

## Data Availability

The data set can be accessed upon request.
